# A Prospective Clinical and Magnetic Resonance Imaging-Based Follow-up Beyond 10 Years After Arthroscopic Matrix-Induced Autologous Chondrocyte Implantation

**DOI:** 10.1177/03635465261459242

**Published:** 2026-07-17

**Authors:** Jay R. Ebert, Peter K. Edwards, Sven Klinken, David J. Wood, Gregory C. Janes

**Affiliations:** † School of Human Sciences (Exercise and Sport Science), University of Western Australia, Perth, Western Australia, Australia; ‡ HFRC Rehabilitation Clinic, Perth, Western Australia, Australia; § School of Allied Health, Curtin University, Perth, Western Australia, Australia; ‖ Perth Radiological Clinic, Perth, Western Australia, Australia; ¶ School of Surgery (Orthopaedics), University of Western Australia, Perth, Western Australia, Australia; # Perth Orthopaedic & Sports Medicine Centre, Perth, Western Australia, Australia; Investigation was performed at the University of Western Australia, Perth, Western Australia, Australia

**Keywords:** arthroscopic, matrix-induced autologous chondrocyte implantation (MACI), clinical outcomes, magnetic resonance imaging

## Abstract

**Background:**

Long-term studies are emerging reporting encouraging outcomes after matrix-induced autologous chondrocyte implantation (MACI). While MACI has historically been undertaken via open arthrotomy, arthroscopic techniques have been reported. However, longer term clinical outcomes and/or graft survivorship after arthroscopically performed MACI are yet to be reported.

**Purpose:**

To present longer term clinical and radiological outcomes in patients after MACI performed arthroscopically on the femoral condyles.

**Study Design:**

Case series; Level of evidence, 4.

**Methods:**

A total of 45 patients were prospectively recruited and underwent arthroscopically performed MACI to the medial femoral condyle (MFC; n = 31; defect size, 1.0-7.7 cm^2^) or lateral femoral condyle (LFC; n = 14; defect size, 1.5-5.0 cm^2^). Outcomes were collected before surgery and at 1, 2, and 5 years as well as at a mean of 13.6 years (range, 11-17 years) after surgery. These included scores for a variety of patient-reported outcome measures (PROMs) including the Knee Injury and Osteoarthritis Outcome Score, Lysholm Knee Score, Tegner Activity Scale, and patient satisfaction. Active knee range of motion as well as peak isokinetic knee extensor and flexor strength were assessed, while magnetic resonance imaging (MRI) was undertaken. The Magnetic Resonance Observation of Cartilage Repair Tissue (MOCART) scoring tool was employed to grade relevant graft parameters, along with an overall MRI composite score.

**Results:**

A significant improvement (*P* < .0001) was observed on most PROMs, with no differences between patients undergoing MACI to the MFC or LFC. Of those assessed at final follow-up, 100% were satisfied with the ability of MACI to relieve their knee pain, while 95% were satisfied with their ability to participate in sport. Active knee flexion range of motion significantly improved (*P* < .0001), as did the knee extensor strength limb symmetry index (*P* < .0001), although no differences were observed based on graft location. Despite encouraging MRI-based scores at final follow-up, a significant deterioration over time was observed for graft infill (*P* = .040), border integration (*P* = .026), surface contour (*P* = .012), tissue structure (*P* = .007), and the overall MRI composite score (*P* = .029), largely as a result of mean changes from 5 years to final follow-up. Apart from better scores for subchondral lamina over the period in LFC grafts, no MRI-based differences were observed based on graft location. One patient demonstrated a graft bed devoid of repair tissue, indicating graft failure, and combined with one additional patient who progressed to total knee arthroplasty, a failure rate of 4.4% was observed.

**Conclusion:**

MACI performed via an arthroscopic approach provided sound clinical outcomes, patient satisfaction, and graft survivorship at a mean 13.6 years after surgery.

Several surgical options are available to treat symptomatic articular cartilage lesions in the knee, including microfracture,^38^ mosaicplasty,^19^ osteochondral autograft transfer system, and autologous chondrocyte implantation (ACI).^6^ While first-^6^ and second-generation^5^ ACI required the injection of cultured chondrocytes under a periosteal or biodegradable collagen membrane, respectively, third-generation ACI (matrix-induced ACI [MACI]) seeds chondrocytes directly onto a type I/III collagen membrane, fixed to subchondral bone with fibrin glue. Encouraging clinical and radiological outcomes at ≥10 years have now been reported.^2,8,12,13,17,29,43^

However, while ACI had traditionally required open arthrotomy for graft implantation, third-generation MACI lends itself to an arthroscopic surgical approach. An arthroscopically performed procedure is less invasive than open arthrotomy and, specific to MACI, has previously demonstrated a reduced risk of complications and reduced hospital stay, permitting an accelerated rehabilitation pathway.^14^ Several arthroscopic techniques for implanting autologous chondrocytes have now been reported, with many technical notes,^15,26,32,35^ case reports,^34^ or small case series^16,21^ and/or small prospective studies presenting early^9,25^ or midterm^11,23^ postoperative clinical outcomes. In literature presenting outcomes to 5 years after the arthroscopic implantation of autologous chondrocytes, one study reported that 90% of patients were satisfied and demonstrated good-excellent repair tissue infill, with a significant preoperative to 5-year postoperative improvement on a variety of patient-reported outcome measures (PROMs) including the Knee Injury and Osteoarthritis Outcome Score (KOOS), Lysholm Knee Score (LKS), and Tegner Activity Scale (TAS).^11^ Kon et al^23^ compared outcomes to 5 years in patients undergoing microfracture versus the arthroscopic implantation of autologous chondrocytes via Hyalograft C, reporting better clinical outcomes (International Knee Documentation Committee objective and subjective scores) and the resumption of sporting activities after the latter. Nonetheless, longer term clinical outcomes, patient satisfaction, and/or graft survivorship after arthroscopically performed MACI are yet to be reported.

The current study sought to present longer term clinical and radiological outcomes in a prospectively recruited cohort of patients who underwent MACI performed via an arthroscopic approach to the femoral condyles. It was hypothesized that (1) a significant improvement on PROMs would be observed from preoperatively to final follow-up, (2) sound graft survivorship would be observed at final follow-up with a low rate (<10%) of graft failure, and (3) no differences in clinical scores or MRI-based outcomes would be observed between those undergoing arthroscopically performed MACI to the medial femoral condyle (MFC) or lateral femoral condyle (LFC).

## Methods

### Participants

**Figure 1.**
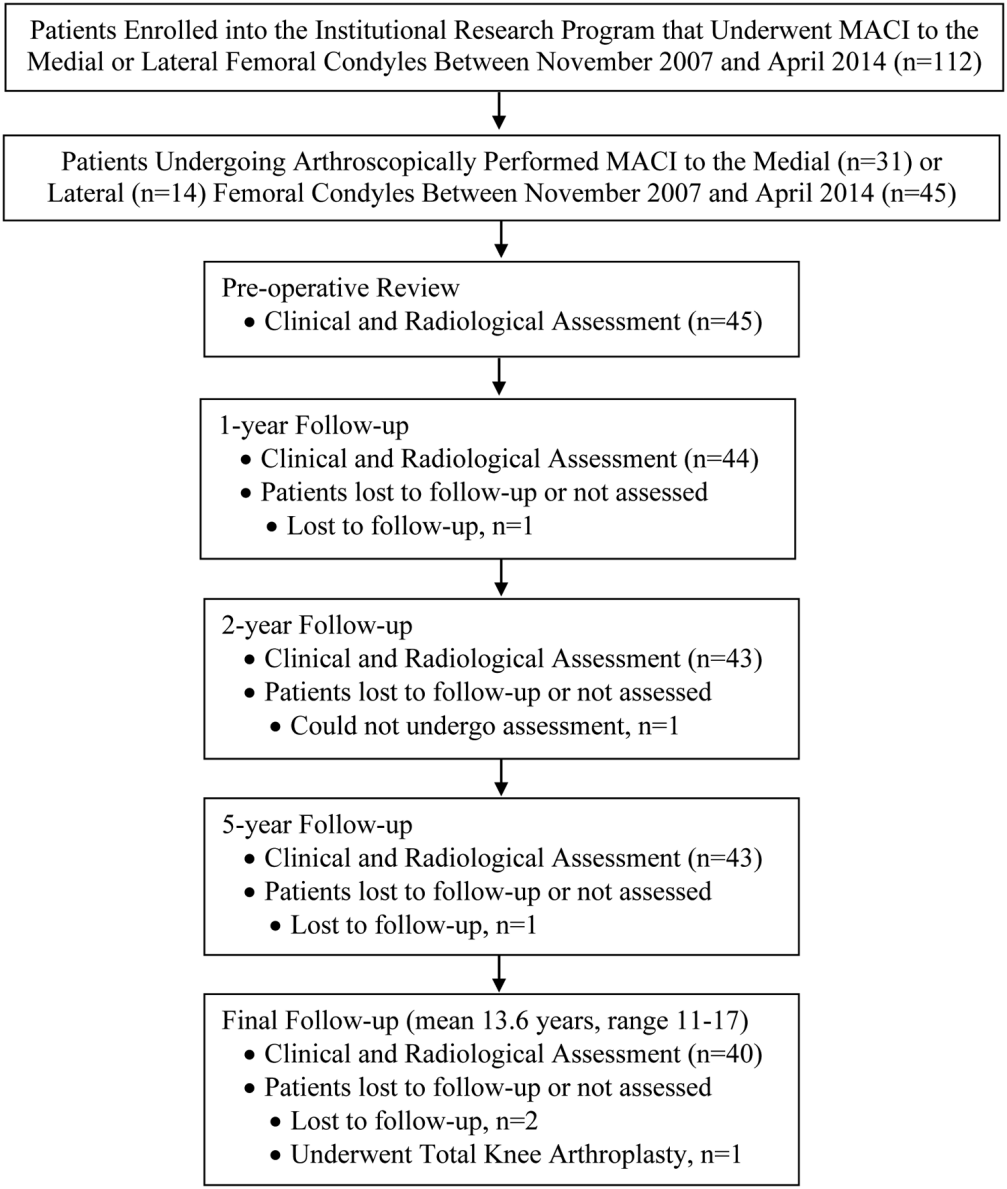
Flowchart demonstrating recruitment, patient review, and loss to follow-up over the assessment period.

Between November 2007 and April 2014, a total of 45 patients were prospectively recruited into an institutional research program (Figure 1), subsequently undergoing MACI performed via an arthroscopic surgical approach to the MFC (n = 31) or LFC (n = 14) by 1 of 2 orthopaedic surgeons (Table 1). All patients provided their written informed consent before study enrollment, and ethical approval was obtained from the relevant hospital ethics committee (No. HPH145), although the study was not recorded in a clinical trials registry.

**Table 1:** Patient Characteristics^*a*^

	Total (n = 45)	MFC (n = 31)	LFC (n = 14)
Final follow-up, y	13.6 (11.0-17.0)	13.5 (11.0-17.0)	14.1 (11.5-17.0)
Age, y	35.1 (18-57)	38.1 (18-57)	33.5 (21-50)
Height, m	1.72 (1.54-1.97)	1.71 (1.54-1.95)	1.73 (1.58-1.97)
Weight, kg	79.1 (46.0-127.9)	78.1 (46.0-118.0)	83.4 (66.0-105.0)
Body mass index, kg/m^2^	26.4 (18.4-34.8)	26.2 (18.4-34.8)	27.6 (23.0-33.1)
Male sex	27 (60.0)	19 (61.3)	8 (57.1)
No. of prior procedures	1.0 (0-5)	0.9 (0-3)	1.2 (0-5)
Duration of symptoms, y	6.1 (1-25)	6.3 (1-25)	5.8 (1-10)
Defect size, cm^2^	2.6 (1.0-7.7)	2.7 (1.0-7.7)	2.4 (1.5-5.0)

a Data are shown as mean (range) or n (%). LFC, lateral femoral condyle; MFC, medial femoral condyle.

All patients exhibited persistent pain and symptoms associated with grade III or IV chondral lesions, assessed with the International Cartilage Repair Society classification system.^7^ These were initially evaluated via baseline magnetic resonance imaging (MRI), and to assess the presence of any other concomitant abnormalities, although they were later confirmed at the time of first-stage arthroscopic biopsy of cartilage. Further inclusion criteria for MACI throughout the designated recruitment period were 15 to 65 years of age, body mass index ≤35 kg/m^2^, and patients who appeared able and willing to follow a structured rehabilitation program. Providing that it was addressed at the time of surgery, patients were surgical candidates for MACI if they presented with ligamentous instability or varus/valgus lower limb malalignment (as indicated by a >3° anatomic tibiofemoral angle). The orthopaedic specialist evaluated the patient for joint malalignment initially on visual inspection and clinical examination, and if further investigation was warranted, the patient was sent for Maquet views. However, concomitant off-loading osteotomy was not required for any patient included in this prospective series. Relevant to the arthroscopic delivery of the MACI graft to the femoral condyles (employing the technique reported), it was deemed that an upper limit of 9 cm^2^ (3 × 3 cm) was reasonable, while the technique was appropriate for the load-bearing femoral condyles and not the anterior aspect of the condyles.

### Surgical Technique

First-stage arthroscopic biopsy and second-stage implantation of the matrix have been previously described.^9,11^ Initially, an arthroscopic surgical procedure was undertaken to harvest healthy articular cartilage from the nonweightbearing trochlear notch or the medial/lateral femoral condylar ridge. At this time, suitability for second-stage arthroscopic implantation was assessed, as was the status of meniscal and ligamentous structures. The cartilage biopsy specimen was sent to the laboratory (Genzyme), where chondrocytes were isolated, cultured over a 4- to 8-week period, and seeded onto a type I/III collagen membrane (ACI-Maix; Matricel) at 3 days before second-stage re-implantation.

Second-stage MACI was undertaken via standard anteromedial and anterolateral portals. Ringer’s lactate solution was employed to irrigate the joint. Initially, the chondral lesion was again inspected and further prepared for implantation by debriding the defect’s base and walls to ensure a well-defined and contained defect. Geometry of the defect was assessed using the end of a graduated arthroscopy probe, with the matrix cut to fit. At this time, the knee was drained of all fluid (converting it to a “dry” arthroscopy setting) and the defect bed dried. The graft was introduced via a large-bore arthroscopic cannula, with an 8-mm inner diameter and no valves (Conmed Linvatec). While further trimming of the graft may have been required to best fit the defect bed, it was then folded away from the defect to permit the introduction of fibrin glue via a 19-gauge needle (Becton Dickinson). The graft was next repositioned in the defect bed, and a Silastic Foley catheter (Cook Medical) was introduced with the balloon inflated with saline to distribute 30 seconds of even pressure over the graft as the glue set. The knee was put through several knee flexion/extension cycles under arthroscopic visualization to ensure graft stability.

### Postoperative Rehabilitation

All patients underwent a coordinated postoperative rehabilitation program that has been previously described.^11^ Initially, early inpatient care consisted of cryotherapy and training in proficient toe-touch ambulation with crutches; continuous passive motion (0°-30°); active ankle movements to encourage lower extremity circulation; and isometric quadriceps, hamstring, and gluteal contractions. After this early period, all patients participated in a supervised outpatient rehabilitation program, consisting of 2 supervised sessions per week over a 12-week period, with ongoing advice and education provided as required up to 12 months. The focus was a graduated increase in weightbearing (with full and unaided weightbearing, as tolerated, permitted from 8 weeks after surgery), knee range of motion (ROM; with full active ROM permitted from 7 weeks after surgery), and progressive exercises designed to improve lower limb strength and functional capacity, with later stage conditioning dictated by individual patient goals.

### Clinical Evaluation

Several PROMs were administered preoperatively (before second-stage MACI) and at 1, 2, and 5 years as well as at final follow-up. These included the KOOS subscales of pain, symptoms, activities of daily living, sport and recreation, and knee-related quality of life; the LKS; the TAS; a visual analog scale to evaluate the frequency and severity of knee pain (0-10); and the physical component summary and mental component summary (MCS) of the 36-Item Short Form Health Survey (SF-36). Furthermore, specifically at final follow-up, the Patient Satisfaction Questionnaire was employed to investigate the overall level of satisfaction as well as each patient’s satisfaction with MACI in relieving knee pain, improving the ability to perform normal daily activities, improving the ability to participate in recreational activities, and improving the ability to participate in sport. A Likert response scale was employed with the following descriptors: very satisfied, somewhat satisfied, somewhat dissatisfied, and very dissatisfied. At all follow-up time points, maximal active knee flexion and extension ROM was assessed on the operated and nonoperated limbs (measured to the nearest degree) as well as peak isokinetic torque of the quadriceps and hamstring muscles. This was evaluated using an isokinetic dynamometer (Isosport), assessing peak concentric knee extension and flexion torque through a range of 0° to 90° of knee flexion at an angular velocity of 90 deg/s. For all efforts, the peak torque value (N·m) was obtained, with the limb symmetry index (LSI) calculated by dividing the peak value on the operated limb by that recorded on the nonoperated limb.

### Radiological Evaluation

High-resolution MRI was undertaken at all follow-up time points using a 1.5- or 3.0-T scanner (Siemens, Philips, or General Electric). The Magnetic Resonance Observation of Cartilage Repair Tissue (MOCART) scoring tool^27,33,40,44^ was employed to assess pertinent parameters of the graft including graft infill, signal intensity, border integration, surface contour, tissue structure, effusion, subchondral lamina, and subchondral bone.^28^ Individual parameters of graft status were assessed as follows: 1 = poor; 2 = fair; 3 = good; and 4 = excellent (with an additional score of 3.5 provided for “graft hypertrophy” in the category of “graft infill”). Furthermore, an overall MRI composite score was also calculated that incorporated these variables.^10,33^ Graft failure was defined as an exposed subchondral bone bed with no evidence of repair tissue or if the patient proceeded to total knee arthroplasty (TKA). The MRI evaluation was performed by an independent, experienced musculoskeletal radiologist.

### Statistical Analysis

While the current sample was limited by the number of patients who underwent MACI performed arthroscopically over the designated recruitment period, an a priori power calculation indicated that at least 19 patients would be required to reveal differences at the 5% significance level, with 90% power and employing a large effect size (0.8), based on preoperative to 2-year postoperative changes on the pain subscale of the KOOS. This was derived from preliminary 2-year data collected in patients undergoing MACI via open arthrotomy, with these outcomes to 10 years after surgery since published.^8^ To allow for attrition, the intention was to therefore recruit at least 25 patients (a further 30%) to ensure adequate power. After recruitment of the required cohort and subsequent ethical approval to continue recruitment, the prospective enrollment of patients continued until which time that MACI ceased to be financially reimbursed in our geographical location (from April 2014).

As reported later, 5 patients underwent meniscectomy concomitantly with their second-stage MACI (with no other concomitant procedures undertaken). Therefore, while no differences were observed (*P* > .05) in relevant patient characteristics (age and body mass index) and injury/surgery history (defect size, duration of symptoms, and prior procedures) between those who did and did not undergo concomitant meniscectomy, no differences (*P* > .05) were also observed in preoperative or minimum 10-year postoperative PROM scores (including KOOS pain, symptoms, and sport and recreation subscales as well as the TAS), or the graft infill score or overall MRI composite score, at final follow-up. Thus, analysis was undertaken in the full cohort that included these 5 patients who underwent concomitant meniscectomy. The mean (along with standard deviation and range) of all clinical and MRI-based measures was calculated and presented. The normality of the distribution of continuous data was assessed and confirmed via the Shapiro-Wilk test. Repeated-measures analysis of variance was employed to evaluate changes in clinical and MRI-based scores over time. Furthermore, clinical and MRI-based scores were compared between those undergoing MACI on the MFC or LFC. Statistical analysis was performed using SPSS software (Version 30.0; IBM), with statistical significance determined at *P* < .05.

## Results

Of the 45 patients who were recruited and underwent MACI via an arthroscopic approach, 40 (88.9%) were available for review at a mean final follow-up of 13.6 years (range, 11-17 years) (Figure 1). Of those who could not be assessed, 4 patients were lost to follow-up, and 1 patient underwent TKA at 8 years after surgery. In the current cohort, 5 patients (11.1%) underwent concomitant meniscectomy at the time of their second-stage MACI, although no other concomitant procedures were undertaken. However, 21 patients (46.7%) had undergone a prior surgical intervention on the ipsilateral knee, including arthroscopy with chondral debridement (n = 14), partial meniscectomy (n = 7), anterior cruciate ligament reconstruction (n = 3), and MACI through open arthrotomy (n = 1). However, no prior (or concomitant) re-alignment procedures were required in the recruited cohort.

A significant clinical improvement (*P* < .05) was observed on all PROMs (apart from the SF-36 MCS) from preoperatively to postoperatively (Table 2), with no significant change (*P* > .05) on any PROM from 5 years to final follow-up. Across the full cohort, mean differences from before surgery to final follow-up were observed for the KOOS pain (26.2-point difference [95% confidence interval (CI), 20.7-30.6]; *d* = 1.6; *P* < .001), symptoms (22.9-point difference [95% CI, 16.9-28.4]; *d* = 1.3; *P* < .001), activities of daily living (18.6-point difference [95% CI, 11.4-22.0]; *d* = 1.0; *P* < .001), sport and recreation (43.3-point difference [95% CI, 36.5-52.3]; *d* = 1.8; *P* < .001), and knee-related quality of life (40.9-point difference [95% CI, 33.5-46.1]; *d* = 1.8; *P* < .001) subscales as well as the LKS (25.0-point difference [95% CI, 20.3-30.9]; *d* = 1.5; *P* < .001). These mean improvements over the minimum 10-year postoperative time frame all exceeded previously reported minimal clinically important difference values in patients undergoing chondrocyte implantation.^30^

**Table 2:** PROM Scores^*a*^

	KOOS					Lysholm	Tegner	SF-36		VAS	
	Pain	Symptoms	ADL	Sport	QOL			PCS	MCS	Frequency	Severity
Before surgery											
MFC	62.5 ± 22.0	68.4 ± 17.4	78.7 ± 20.9	35.3 ± 22.7	33.2 ± 17.4	55.4 ± 17.6	2.6 ± 1.2	38.0 ± 8.7	54.3 ± 7.1	6.2 ± 2.6	5.8 ± 2.3
LFC	67.3 ± 12.5	60.9 ± 17.0	78.0 ± 15.5	33.6 ± 21.3	32.6 ± 11.7	66.1 ± 14.0	3.0 ± 0.7	46.3 ± 8.6	46.6 ± 10.2	6.1 ± 2.2	4.8 ± 2.0
1 year											
MFC	85.8 ± 9.3	87.9 ± 8.4	94.1 ± 8.0	58.8 ± 25.2	61.7 ± 16.6	83.3 ± 11.2	5.5 ± 1.6	48.5 ± 8.0	56.5 ± 7.9	1.9 ± 1.8	1.9 ± 1.5
LFC	80.5 ± 15.2	84.4 ± 9.0	89.7 ± 12.1	60.7 ± 25.2	52.2 ± 19.5	78.0 ± 9.0	4.7 ± 1.3	48.1 ± 5.7	55.5 ± 5.9	2.6 ± 1.8	2.6 ± 1.7
2 years											
MFC	88.0 ± 10.4	87.6 ± 8.9	94.7 ± 8.1	66.3 ± 26.6	67.5 ± 20.6	88.2 ± 7.0	5.4 ± 1.4	50.2 ± 7.8	56.8 ± 7.3	1.9 ± 2.3	1.7 ± 1.6
LFC	89.0 ± 8.1	87.3 ± 5.7	94.2 ± 5.5	68.2 ± 23.2	58.9 ± 19.4	83.9 ± 9.3	4.9 ± 1.4	50.2 ± 5.8	53.9 ± 4.6	2.5 ± 1.7	2.5 ± 1.5
5 years											
MFC	90.2 ± 8.5	88.6 ± 9.5	94.9 ± 6.9	76.8 ± 25.0	67.7 ± 24.9	85.0 ± 14.3	5.8 ± 1.9	51.3 ± 6.4	55.1 ± 9.4	2.1 ± 2.4	1.9 ± 1.6
LFC	94.1 ± 5.8	85.1 ± 8.6	97.1 ± 4.3	81.6 ± 20.4	74.5 ± 22.7	86.8 ± 19.2	5.6 ± 1.3	53.7 ± 3.5	54.5 ± 6.1	1.8 ± 1.6	1.8 ± 1.2
>10 years											
MFC	89.6 ± 10.3	87.8 ± 8.6	95.3 ± 6.7	77.0 ± 17.5	71.3 ± 16.3	82.5 ± 12.0	5.1 ± 1.8	49.8 ± 7.4	52.6 ± 9.9	1.7 ± 1.9	1.7 ± 1.6
LFC	92.9 ± 6.3	91.7 ± 6.6	97.6 ± 3.6	78.8 ± 16.4	76.5 ± 16.2	88.2 ± 10.8	4.9 ± 1.1	52.7 ± 4.6	49.9 ± 9.9	1.3 ± 1.7	1.1 ± 1.3
*P* value											
Time effect	<.0001	<.0001	<.0001	<.0001	<.0001	<.0001	<.0001	<.0001	.354	<.0001	<.0001
Group effect	.729	.431	.915	.347	.983	.556	.471	.108	.094	.917	.957
Interaction effect	.261	.069	.244	.499	.094	.797	.665	.199	.683	.213	.473

a Data are shown as mean ± SD, with the analysis of variance results reporting the time, group, and group × time interaction for PROMs based on graft location (MFC vs LFC). ADL, activities of daily living; KOOS, Knee Injury and Osteoarthritis Outcome Score; LFC, lateral femoral condyle; MCS, mental component summary; MFC, medial femoral condyle; PCS, physical component summary; PROM, patient-reported outcome measure; QOL, quality of life; SF-36, 36-Item Short Form Health Survey; VAS, visual analog scale.

Furthermore, there were no differences between patients undergoing MACI to the MFC or LFC (Table 2). Of the 40 patients who completed the Patient Satisfaction Questionnaire at final follow-up, 100.0% were satisfied with the ability of MACI to relieve their knee pain, while 95.0% were satisfied with the improvement in their ability to participate in sport (Table 3). Additionally, of the 39 patients who were satisfied with their ability to participate in recreational activities, 31 (79.5%) were “very satisfied” (vs “somewhat satisfied”). Of the 38 patients who were satisfied with their ability to participate in sport, only 22 (57.9%) were “very satisfied” (vs “somewhat satisfied”).

**Table 3:** Satisfaction Ratings (n = 40)^*a*^

	Relieving Knee Pain	Improving Ability to Undertake Activities of Daily Living	Improving Ability to Participate in Recreational Activities	Improving Ability to Participate in Sport	Overall Satisfaction
Very satisfied	31	34	31	22	30
Somewhat satisfied	9	6	8	16	9
Somewhat dissatisfied	0	0	1	2	1
Very dissatisfied	0	0	0	0	0
Satisfied overall	40	40	39	38	39

a Data are shown as No. “Satisfied overall” represents a combination of “somewhat satisfied” and “very satisfied.”

Active knee flexion ROM on the operated limb significantly improved over time, as did the knee extensor strength LSI (Table 4). No statistically significant differences were seen in knee ROM measurements or strength LSIs between patients undergoing MACI to the MFC or LFC (Table 4), and mean isokinetic strength LSIs were around 100% at final follow-up (Table 4).

**Table 4:** Active Knee Range of Motion^*a*^

	Maximal Knee Flexion, deg		Maximal Knee Extension, deg		Knee Extensor Strength LSI	Knee Flexor Strength LSI
	Operated	Nonoperated	Operated	Nonoperated		
1 year						
MFC	142.7 ± 6.2	142.5 ± 6.5	−1.3 ± 1.7	−1.5 ± 1.9	88.5 ± 15.9	99.3 ± 16.6
LFC	143.0 ± 3.1	144.8 ± 3.3	−2.1 ± 2.3	−2.6 ± 2.1	87.0 ± 17.5	94.9 ± 17.3
2 years						
MFC	143.4 ± 6.1	143.5 ± 6.6	−1.6 ± 1.6	−1.5 ± 1.6	92.4 ± 13.8	98.8 ± 20.5
LFC	143.3 ± 3.9	143.6 ± 4.1	−2.2 ± 2.1	−2.3 ± 2.1	94.7 ± 16.0	96.3 ± 12.1
5 years						
MFC	144.4 ± 6.7	144.0 ± 7.6	−0.9 ± 1.5	−1.0 ± 1.3	96.8 ± 11.7	101.1 ± 11.4
LFC	147.4 ± 5.3	147.4 ± 6.8	−2.0 ± 1.7	−2.0 ± 1.7	97.8 ± 10.5	96.7 ± 11.1
>10 years						
MFC	145.7 ± 7.1	146.3 ± 7.7	−0.9 ± 1.7	−1.1 ± 1.7	101.0 ± 13.7	100.0 ± 14.0
LFC	146.4 ± 5.6	146.5 ± 6.6	−1.8 ± 2.1	−2.1 ± 2.2	102.5 ± 19.2	96.1 ± 8.5
*P* value						
Time effect	<.0001	.002	.095	.155	<.0001	.358
Group effect	.750	.585	.061	.059	.569	.280
Interaction effect	.356	.763	.885	.975	.071	.664

a Data are shown as mean ± SD, with the analysis of variance results reporting the time, group, and group × time interaction for functional outcome variables based on graft location (MFC vs LFC). Negative values indicate hyperextension (or extension beyond neutral). LFC, lateral femoral condyle; LSI, limb symmetry index; MFC, medial femoral condyle.

**Figure 2.**
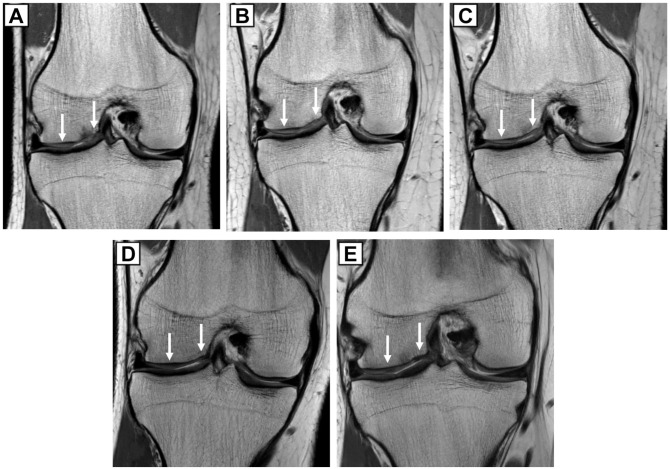
Proton density fast spin echo magnetic resonance imaging of (A) a chondral defect preoperatively on the lateral femoral condyle (between white arrows) as well as the matrix-induced autologous chondrocyte implantation (MACI) graft at (B) 1 year, (C) 2 years, (D) 5 years, and (E) 17 years after surgery.

Mean overall MRI composite scores of 2.96 and 2.97 (of a possible 4) at final follow-up were observed for those undergoing arthroscopic MACI to the MFC and LFC, respectively (Table 5). A statistically significant deterioration was observed in graft infill, border integration, surface contour, tissue structure, and the overall MRI composite score over the full postoperative follow-up period (Table 5). However, while no statistically significant change (*P* > .05) in MRI-based scores was observed up until 5 years after surgery, these changes were a result of the statistically significant deterioration in scores from 5 years to final follow-up. The LFC group demonstrated consistently better scores for subchondral lamina over the period, although apart from a significant interaction for signal intensity, no other MRI-based differences were observed between patients who underwent MACI to the MFC or LFC (Table 5). Figure 2 shows a preoperative chondral defect on the LFC and graft progression after MACI in a recruited patient. One patient included in the current cohort had previously undergone MACI through open arthrotomy to the same graft location in October 2002. After initial MACI in 2002, this patient demonstrated sound clinical and MRI-based outcomes at 5 years after surgery, with a recurrence of symptoms at 9 years after first surgery. The decision was made to reoperate and undertake MACI through an arthroscopic technique in November 2012, and 12-year follow-up demonstrated a “very satisfied” patient; scores of 94.2, 82.1, 75.0, and 7 for the KOOS pain, KOOS symptoms, KOOS sport and recreation, and TAS, respectively; and scores of 3.00 and 3.15 for graft infill and the overall MRI composite score, respectively.

**Table 5:** MRI Assessment Scores^*a*^

	Graft Infill	Signal Intensity	Border Integration	Surface Contour	Tissue Structure	Subchondral Lamina	Subchondral Bone	Effusion	Overall MRI Composite Score
1 year									
MFC	3.50 ± 0.45	3.07 ± 0.81	3.00 ± 1.01	3.14 ± 1.08	3.32 ± 0.82	3.57 ± 0.63	2.96 ± 1.00	3.61 ± 0.57	3.27 ± 0.53
LFC	3.25 ± 1.05	2.43 ± 0.76	2.79 ± 1.12	2.71 ± 1.27	3.07 ± 1.14	3.71 ± 0.47	2.71 ± 0.73	3.71 ± 0.61	2.96 ± 0.76
2 years									
MFC	3.61 ± 0.34	2.86 ± 0.56	3.14 ± 0.83	3.27 ± 0.98	3.36 ± 0.85	3.64 ± 0.49	2.86 ± 1.04	3.68 ± 0.57	3.30 ± 0.36
LFC	3.55 ± 0.50	3.30 ± 0.48	3.50 ± 0.71	3.80 ± 0.42	3.80 ± 0.42	3.80 ± 0.42	2.60 ± 1.07	3.80 ± 0.42	3.52 ± 0.32
5 years									
MFC	3.40 ± 0.69	2.81 ± 0.49	2.88 ± 1.11	2.77 ± 1.21	3.12 ± 0.99	3.54 ± 0.58	2.81 ± 1.13	3.77 ± 0.43	3.05 ± 0.58
LFC	3.54 ± 0.89	3.00 ± 0.74	2.92 ± 1.00	2.67 ± 1.07	3.17 ± 1.11	3.83 ± 0.39	2.50 ± 1.09	3.83 ± 0.39	3.21 ± 0.69
>10 years									
MFC	3.13 ± 0.47	2.75 ± 0.44	2.67 ± 0.96	2.42 ± 1.21	2.42 ± 1.17	3.17 ± 0.56	2.83 ± 1.09	3.75 ± 0.44	2.96 ± 0.40
LFC	3.00 ± 0.74	2.83 ± 0.72	2.42 ± 1.08	2.50 ± 1.00	2.42 ± 1.24	3.58 ± 0.51	2.67 ± 0.98	3.75 ± 0.45	2.97 ± 0.58
*P* value									
Time effect	.040	0.125	.026	.012	.007	.069	.740	.631	.029
Group effect	.708	.276	.491	.524	.463	.019	.469	.693	.314
Interaction effect	.417	.048	.438	.801	.730	.770	.836	.621	.937

a Data are shown as mean ± SD, with the analysis of variance results reporting the time, group, and group × time interaction for MRI-based outcome variables based on graft location (MFC vs LFC). LFC, lateral femoral condyle; MFC, medial femoral condyle; MRI, magnetic resonance imaging.

**Figure 3.**
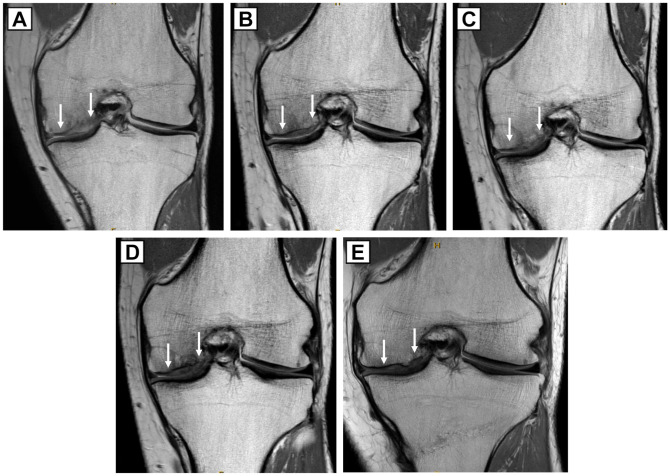
Proton density fast spin echo magnetic resonance imaging of a matrix-induced autologous chondrocyte implantation (MACI) graft (between white arrows) to the medial femoral condyle of the same patient at (A) 3 months, (B) 1 year, (C) 2 years, (D) 5 years, and (E) 16 years after surgery (after undergoing high tibial osteotomy at 5.5 years after MACI).

No early postoperative complications were observed, such as wound infections, hematomas, or deep vein thrombosis. Secondary procedures (after MACI) were undertaken in 2 patients who underwent arthroscopic surgery and partial meniscectomy, 2 patients who underwent graft debridement because of symptoms associated with a hypertrophic graft, and 1 patient who underwent high tibial osteotomy at 5.5 years after MACI because of persistent symptoms (Figure 3). While one patient demonstrated a graft bed devoid of repair tissue, indicating graft failure (who also demonstrated failure at 5 years), another patient underwent TKA at 8 years after MACI (4.4% [2/45] failure rate).

## Discussion

The most important findings of the current study were that arthroscopically performed MACI resulted in improved (and sustained) clinical scores over a mean 13.6-year postoperative period, along with a high rate of patient satisfaction. Sound graft survivorship was observed over the longer term follow-up period, with a low graft failure rate. Furthermore, clinical and MRI-based outcomes were largely similar between patients undergoing MACI on the MFC or LFC.

All PROM scores (apart from the SF-36 MCS score) significantly improved over time, which was in support of the first hypothesis. Furthermore, while patient-reported clinical scores generally peaked around 5 years after surgery, no significant change was observed from 5 years to final follow-up (mean, 13.6 years). While longer term results after arthroscopically performed MACI are lacking, other studies reporting ≥10-year clinical outcomes after MACI performed through open arthrotomy have reported similar postoperative improvements.^2,8,12,13,17,29^ Of interest, TAS scores in the current cohort indeed reduced from 5 years after surgery to final follow-up as expected, although even at a mean 13.6 years after surgery, the mean TAS scores were 5.1 and 4.9 for the MFC and LFC groups, respectively. These were also similar to other studies reporting on the postoperative TAS score at longer term follow-up.^8,17^ These sustained scores over the mean 13.6-year period were further supported by the high satisfaction levels observed, especially with respect to pain relief, undertaking activities of daily living, and participating in recreational activities. However, it is worth reporting that in investigating satisfaction with the ability to participate in recreational versus sporting activities, the response of “very satisfied” versus “somewhat satisfied” was greater for recreational (79.5% “very satisfied”) versus sport (57.9% “very satisfied”) activities. This may be related to the mean 13.6-year (range, 11-17 years) follow-up in a cohort with a mean age of 35.1 years (range, 18-57 years) at the time of their arthroscopic MACI procedure. Nonetheless, future studies should seek to accurately report patient satisfaction in various domains of recovery (ie, activities of daily living, recreational and sporting activities) rather than simply a generic satisfaction response.

An improvement in the knee extensor strength LSI was observed over the postoperative timeline, reflecting inadequate strength recovery at 1 year after surgery and a gradual improvement over the first 5 years. The importance of lower limb strength restoration has been previously reported in other studies and after different lower limb procedures, along with its link with a successful return to physical activity.^1,3,22,24,39^ The mean knee extensor and flexor strength LSIs observed in the current study were generally around 100% at 5 years and final follow-up. While not specifically assessed as part of the current study, this restoration of lower limb strength, combined with the ability to maintain an active lifestyle, likely contributes to the patient satisfaction reported.

It was clear in the current study that MRI-based scores peaked at 2 years after surgery, with a gradual decline from 2 to 5 years and from 5 years to final follow-up. It is important to note that despite the decline, encouraging MRI-based scores (particularly graft infill and the overall MRI composite score) were still observed at final follow-up, and only 2 patients (one with MRI demonstrating no repair tissue and one who progressed to TKA) essentially failed. This was in support of the second hypothesis. In other studies that have reported MOCART scores at ≥10 years after third-generation MACI,^8,12,13^ MRI-based outcomes appear comparable to those observed in the current study, despite the current study reporting a longer mean follow-up. However, it should also be reiterated that 2 further patients underwent secondary arthroscopic surgery after their primary MACI to debride a hypertrophic and symptomatic graft, although both of these patients were satisfied with their outcomes at final follow-up.

Despite the relatively small patient cohort, a comparison was made between those who underwent arthroscopically performed MACI on the MFC or LFC. No differences were observed in PROM scores, knee ROM, or isokinetic strength measurements, as well as the majority of MRI-based outcomes, over the postoperative period. This was in support of the third hypothesis. Of interest, across the postoperative period, the MRI-based outcome for subchondral lamina was consistently better in the LFC group, and while no differences in defect size were observed between those undergoing arthroscopic MACI to the MFC or LFC, this may be more attributable to the general increase in loading borne by the medial (vs lateral) knee compartment in walking and trivial daily weightbearing activities.^4^ Nonetheless, the mean 13.6-year graft infill and overall MRI composite scores were similar based on defect location.

As mentioned above, longer term outcomes after arthroscopically performed MACI are lacking, although the clinical and MRI-based results reported at a mean 13.6 years in the current study are similar to longer term (≥10 years) outcomes reported previously in patients undergoing MACI via open arthrotomy.^2,8,12,13,17,29^ However, other surgical options are routinely employed for the treatment of symptomatic knee chondral defects, with studies also reporting longer term outcomes. Gobbi et al^18^ presented outcomes at a mean of 15.1 years (range, 10-20 years) in patients who underwent microfracture, reporting good outcomes at 2 years, with a deterioration to 5 years and degenerative knee changes at longer term follow-up. They also reported better outcomes in smaller chondral lesions (≤4 cm^2^) and younger (≤30 years) patients. While Ulstein et al^41^ reported no differences between microfracture and osteochondral autograft transfer system at a median 9.8 years after surgery, Solheim et al^36,37^ reported better 10-year outcomes in patients undergoing mosaicplasty versus microfracture, despite similar patient-reported outcomes at 15 to 18 years. A more recent study reported encouraging 10-year outcomes in patients who underwent autologous matrix-induced chondrogenesis, which were also superior to microfracture.^42^

We acknowledge some limitations in this study. First, it consists of a prospectively recruited cohort, with a relatively small sample size, and is a reflection of those who underwent MACI performed arthroscopically over the designated period. Furthermore, there was no control or comparative group included, although comparisons of outcomes to existing literature can be made. Several other cartilage repair options exist, and some of these are certainly suitable for smaller chondral lesions (≤4 cm^2^),^19,38^ although throughout the designated recruitment period, MACI was easily accessible and routinely employed within our geographical location and was also considered the gold-standard treatment method at that time. The current study was able to evaluate 40 of 45 patients at a mean 13.6-year follow-up, and the inability to include clinical scores in the 11.1% who were not assessed at final follow-up (although one of these underwent TKA and was reported) may slightly skew the longer term reported outcomes. It should be noted that 2 of these patients were lost to follow-up before 5-year follow-up, while the other 2 patients who were lost between the 5-year and mean 13.6-year time points were highly satisfied and presented with good MRI-based outcomes at 5 years. Finally, the MOCART scoring tool was employed to evaluate graft status, as opposed to any widespread intra-articular knee changes. Whole-organ osteoarthritic knee scoring tools,^20,31^ particularly over a longer time period and in comparison to a normative cohort that is not undergoing knee surgery, may be considered in future studies. It is also acknowledged that a relatively large number of outcome variables (patient-reported, clinical, and radiological) were examined across the study. No statistical correction for multiple comparisons was applied across outcome variables; therefore, findings should be interpreted with appropriate caution.

MACI undertaken via the reported arthroscopic technique demonstrated improved (and sustained) clinical scores over a mean 13.6-year postoperative period, along with a high rate of patient satisfaction. Sound graft survivorship was observed over the longer term follow-up period, with a low graft failure rate. Clinical and MRI-based outcomes were largely similar between patients undergoing arthroscopically performed MACI on the MFC or LFC.
